# Genetic polymorphisms of the 5’ untranslated regions of the *HSP70* gene in Indonesian cattle populations

**DOI:** 10.14202/vetworld.2022.168-172

**Published:** 2022-01-27

**Authors:** Peni Wahyu Prihandini, Almira Primasari, Aryogi Aryogi, Muchamad Luthfi, Dwi Nur Happy Hariyono

**Affiliations:** 1 Beef Cattle Research Institute of Grati, Pasuruan 67183, Indonesia; 2 Department of Animal Science, Faculty of Agriculture, Universitas Khairun, Ternate 97719, Indonesia.

**Keywords:** cattle, heat shock protein, heat stress, polymorphism, thermotolerance

## Abstract

**Background and Aim:**

Heat shock proteins (HSPs) are a group of proteins that play a significant role in protecting cells against cellular stress. *HSP70* is a conserved, sensitive, and abundant gene associated with heat stress’s physiological adaptability. The objective of this study was to reveal the polymorphisms of the partial sequences of the *HSP70* gene (5’ untranslated region [UTR]) in seven cattle populations in Indonesia.

**Materials and Methods:**

Polymerase chain reaction products (551 bp) of the *HSP70* gene amplified from 102 animals representing seven cattle populations (Bali, Belgian Blue x Peranakan Ongole [PO] cross, Galekan, Jabres, Madura, PO, and Rambon) were sequenced by DNA sequencing method.

**Results:**

Fourteen single-nucleotide polymorphisms (SNPs), generally found at a low frequency, were detected. Among these SNPs, only 1117G>A, 1125A>C, and 1204T>C were polymorphic in all the analyzed breeds. A Chi-square test showed that the majority of the loci were in Hardy–Weinberg equilibrium (p*>*0.05). Varying levels of observed (0.050-0.571) and expected heterozygosity (0.049-0.500) were noted. The polymorphism information content values (0.048-0.375) indicated that the SNPs in the *HSP70* gene showed low-to-moderate polymorphism in the studied populations. Thirty-six haplotypes were defined according to the identified SNPs, of which haplotype Hap5 (CGACGAGAGTGTCC) and Hap4 (CGACGAGAGTGCCC) were generally dominant in the studied samples. The phylogenetic tree showed a close relationship between Bali and Rambon cattle and between Galekan and Jabres cattle, while the Belgian Blue x PO crossbred cattle were farther apart.

**Conclusion:**

The polymorphisms in the 5’ UTR of the *HSP70* geneidentified in this study should be further investigated in a larger population to unravel the association between the SNPs and thermotolerance in Indonesian local cattle populations.

## Introduction

Cattle farming plays a significant economic and social role for rural households in developing countries like Indonesia. Many local cattle breeds are reared by livestock farmers, especially in the rural areas with low-input farming systems, contributing approximately 90% of total cattle production in Indonesia [1]. However, cattle often suffer from various thermal stresses in tropical regions like Indonesia, such as heat stress. Heat stress is a significant threat to the viability and sustainability of cattle production and has been associated with reducing productive and reproductive performances, leading to economic losses. It is important to exploit the genetic variation underlying thermotolerance traits to reduce heat stress on cattle production.

Certain genes are beneficial in heat stress tolerance. These genes include heat shock proteins (HSPs), a group of proteins that play a major role in providing thermotolerance in cells and protecting cells against apoptosis during injury and cellular stress [2]. HSPs are released in cells in response to several environmental and oxidative stresses [3]. Most HSPs functionally act as molecular chaperones by selectively recognizing and binding non-native proteins, preventing irreversible aggregation under physiological and stress conditions [4]. The HSPs family consists of HSP110, HSP100, HSP90, HSP70, HSP60, HSP40, HSP10, and small HSP. Among them, *HSP70* is the most conserved, sensitive, and abundant gene associated with stress [5,6]. HSP70 is produced by the *HSP70* gene, characterized by a single exon. The open reading frame of this gene is approximately 1926, and its protein consists of 641 amino acids. Heat shock genes are activated by stressful stimuli, forming HSPs [7].

Abundant genetic variations in the HSP70 gene sequences have been reported in some livestock animals, such as in cattle [8-11], goats [12-14], and chickens [15]. *HSP70* gene polymorphisms are genetically associated with milk production traits in Frieswal crossbred cattle [8], blood biochemical parameters in Holstein cattle [16], and heat thermotolerance (HTC) in Tharparkar cattle [9]. Studies reported abundant polymorphisms in the 5’ untranslated region of the *HSP70* in cattle [10,11,17]. A single-nucleotide polymorphism (SNP) at 1128G>T in the 5’ UTR of the *HSP70* gene is associated with an increased ability of peripheral blood mononuclear cells (PBMCs) to respond to heat shock [18]. At present, there is a paucity of literature on the identification of the *HSP70* gene polymorphisms in Indonesian cattle [11]. Good knowledge of the genetic polymorphisms in certain genes will be helpful in determining the SNPs responsible for important economic traits [19,20].

Therefore, this study aimed to identify the genetic polymorphisms of the 5’ UTR of the *HSP70* gene in various cattle populations in Indonesia, which may be helpful in the genetic improvement of the cattle for heat tolerance traits.

## Materials and Methods

### Ethical approval

The experimental procedures were approved by the Institutional Animal Care and Use Committee of the Indonesian Agency for Agricultural Research and Development (Balitbangtan/Lolitsapi/Rm/09/2020).

### Study period and location

The study was conducted from January to December 2020. All the breeds analyzed were obtained from the Beef Cattle Research Institute (BCRI) of Grati, East Java. In addition, the Galekan, Rambon, and Jabres cattle were also collected from Trenggalek of East Java Province, Banyuwangi of East Java Province, and Brebes of Central Java Province, respectively. The samples were processed at the Laboratory of Animal Molecular Genetics, BCRI.

### Animals and sampling

A total of 102 animals from several cattle populations raised in Indonesia were used. Breeds analyzed included Bali (n=16), PO (n=20), Belgian Blue x PO cross (BBPO; n=7), Galekan (n=8), Jabres (n=18), Madura (n=17), and Rambon (n=16). Blood samples taken from the jugular vein were collected in 3 mL tubes containing EDTA as an anticoagulant and stored at 4°C before being analyzed.

### DNA extraction and polymerase chain reaction (PCR) amplification

Genomic DNA was extracted from the blood samples using a gSYNC™ DNA extraction kit (Geneaid, New Taipei City, Taiwan), following the manufacturer’s instructions. A specific pair of primers, HSP70_F, 5’-GTCGCCAGGAAACCAGAGAC-3’ and HSP70_R, 5’- GGAACACCCCTACGCAGGAG-3’ [21], was used to amplify the 551 bp of the bovine *HSP70* gene (GenBank Accession No. M98823). The PCR reaction, which consisted of 2 mL template DNA (10-100 ng), 0.5 mL of each primer (0.25 mM), 12.5 mL PCR kit diluent (2x My Taq HS Red Mix gSYNCTMPCR Kit-Bioline-London), and 9.5 mL ddH2O for a total volume of 25 mL, was conducted using SensoQuest (Germany). The thermal cycle profile included an initial denaturation at 95°C for 5 min, followed by 35 cycles of 94°C for 45 s, 60°C for 45 s, and 72°C for 60 s, with a final extension step at 72°C for 5 min. The PCR products were confirmed by electrophoresis on a 1.5% agarose gel before sequencing using an ABI 3730xl genetic analyzer (Applied Biosystems, Foster City, CA, USA).

### Statistical analysis

The *HSP70* gene sequencing results were arranged, edited, and aligned using BioEdit software [22]. Allele and genotype frequencies, observed and expected heterozygosity, and Chi-square (*χ*^2^) tests were determined usingPOPGENE 1.32 software [23]. The polymorphism information content (PIC) was also calculated. Haplotype combination for the fourteen polymorphic sites was estimated usingDnaSP version 6.12.01 software [24]. A neighbor-joining (NJ) phylogenetic tree was constructed usingMEGA version 5.0 [25].

## Results

### Polymorphisms and genetic diversity

A 551 bp fragment of the 5’ UTR of the bovine *HSP70* gene was amplified and sequenced for 102 animals from seven cattle populations in Indonesia. Consequently, 14 SNPs, namely, nt 1036C>T, 1045G>A, 1058A>G, 1069C>T, 1076G>A, 1096A>G, 1117G>A, 1125A>C, 1128G>T, 1134T>C, 1164G>T, 1204T>C, 1255C>T, and 1262C>T, were identified across the pooled samples (Supplementary data can be available from the corresponding author). In general, the SNPs found in this study were at a low frequency. Only three loci, namely, nt 1117G>A, 1125A>C, and 1204T>C, were polymorphic in all the analyzed breeds.

The Chi-square (*χ*^2^) tests showed that the frequencies of the genotypes were in agreement with the Hardy–Weinberg equilibrium (p*>*0.05), except for nt 1096A>G in Bali cattle, nt 1128G>T in BBPO cattle, nt 1117G>A in Jabres cattle, nt 1045G>A, 1096A>G, 1117G>A, and 1125A>C in Madura cattle, nt 1076G>A in PO cattle, and 1262C>T in Rambon cattle. The observed and expected heterozygosity varied from 0.050 to 0.571 and from 0.049 to 0.500, respectively. To obtain information regarding the 14 identified SNPs, the PIC value for each locus was estimated as per the classification: PIC value<0.25, low polymorphism; 0.25≤PIC value ≤0.5, intermediate polymorphism; and PIC value >0.5, high polymorphism [26]. The PIC values ranged from 0.048 to 0.375, indicating that the SNPs in the *HSP70* gene exhibited low-to-moderate polymorphism in the studied populations.

### Haplotype and phylogenetic analysis

**Figure-1:**
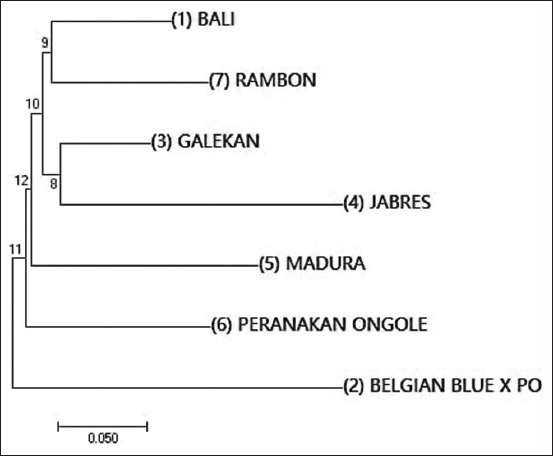
Phylogenetic relationship of the *HSP70* gene sequences among Indonesian cattle populations.

Based on the 14 SNPs, 36 haplotypes were identified in the experimental samples (Supplementary data can be available from the corresponding author). The two most common haplotypes in all breeds were Hap5 (CGACGAGAGTGTCC) and Hap4 (CGACGAGAGTGCCC), with frequencies of 0.25 and 0.22, respectively. The frequencies of the remaining haplotypes ranged from 0.01 to 0.05. Nei’s genetic distance ranged from 0.138 (between Galekan and Bali cattle) to 0.382 (between Jabres and BBPO cattle) (Table-1). As shown in Figure 1, the phylogenetic tree of the seven cattle populations constructed from the matrix genetic distance indicated that Bali and Rambon cattle were closer, followed by Galekan, Jabres, Madura, and PO cattle, while BBPO crossbred cattle were farther apart.

**Table-1:** Pairwise genetic distance between the seven cattle populations based on the *HSP70* SNPs.

Population	Bali	BBPO	Galekan	Jabres	Madura	PO	Rambon
Bali							
BBPO	0.257						
Galekan	0.138	0.266					
Jabres	0.243	0.382	0.208				
Madura	0.207	0.321	0.193	0.294			
PO	0.192	0.295	0.164	0.276	0.233		
Rambon	0.154	0.294	0.151	0.253	0.228	0.206	

PO=Peranakan Ongole cattle, BBPO=Belgian Blue x PO cattle

## Discussion

Heat stress has become a significant issue in climate change because it directly impacts the adaptability and survivability of farm animals to thermal assault. The HSP genes such as *HSP70* and *HSP90* are members of HSPs subfamily (molecular chaperone families) known to be highly expressed under stressful environmental and physiological conditions [27]. This study analyzed the polymorphisms of the 5’ UTR of the *HSP70* gene in the seven cattle populations in Indonesia. A total of 14 SNPs were found after direct sequencing and alignment of all the sequences from 102 samples. These SNPs can be further compared with those reported in published studies.

In mammals like cattle, the *HSP70* gene was extensively studied. All the SNPs found in this study have been reported previously in Pasundan cattle, except for nt 1076G>A [11]. This SNP, however, was found in South African Nguni crossbred cattle [10] and in this study. Interestingly, SNP at nt 1164G>T identified in this study was also found in Pasundan cattle from Indonesia [11], but this SNP was absent in South African Nguni crossbred cattle [10]. Öner *et al*. [17] sequenced the 5’ UTR of the *HSP70* gene in native Turkish and Holstein Friesian cattle and found an abundance of polymorphisms. Among the 14 SNPs identified in this study, only SNPs at nt 1036C>T, nt 1069C>T, and nt 1262C>T were absent in Turkish and Holstein Friesian cattle [17]. Similar to the results of this study, the majority of SNPs found in the 5’ UTR of the *HSP70* gene is at low frequency [11,17]. Several SNPs previously found in the *HSP70* gene, including nt 1045G>A, 1117G>A, 1125A>C, 1128G>T, 1134T>C, and 1204T>C [8,17,18], were also observed in Indonesian cattle. It is noteworthy that the 5’ UTR of the *HSP70* gene contains considerable polymorphisms.

Some previous studies showed the relationship between polymorphisms in the *HSP70* gene and particular traits of interest. For instance, Bhat *et al*. [9] found a significant association between *HSP70* gene polymorphism and HTC in Tharparkar cattle, while Hu *et al*. [28] reported that two SNPs (SNP-42^−^ and SNP-205^+^) are causative polymorphisms involved in the modulation of HSP70 promoter activity and might contribute to the association between the *HSP70* gene and triiodothyronine and thyroxine levels in Sanhe cattle. Among many SNPs found in the 5’ UTR of the *HSP70* gene, SNP at nt 1128G>T is associated with an increased ability of PBMC to respond to heat shock in terms of gene expression and synthesis of HSP70 and cell viability [18]. This SNP was also found in this study; thus, it is necessary to investigate its association with HTC in Indonesian cattle. The SNPs at nt 1045G>A, 1134T>C, and 1204T>C, which were identified in this study, have been reported to be associated with the serum concentration of T3 and IGF-I and body condition [29,30]. Among these SNPs, only nt 1204T>C was observed in all the breeds investigated in this study, while the nt 1045G>A and 1134T>C were identified in all the breeds, except in Jabres cattle (both SNPs) and PO cattle (nt 1134T>C). Therefore, the SNPs identified in this study may be helpful in the association analysis with certain traits of interest, especially HTC in cattle.

All of the SNPs found in this study were used to define the haplotype of the *HSP70* gene in Indonesian cattle. Thirty-six haplotypes were identified, of which haplotypes Hap4 and Hap5 represent the most frequent haplotypes of the *HSP70* gene in the studied populations. Based on the matrix genetic distance, a NJ tree was constructed to show the phylogenetic relationship of the studied populations (Figure-1). The NJ tree indicated that Bali and Rambon cattle were genetically close to Galekan and Jabres, followed by Madura and PO cattle, while BBPO crossbred cattle was farther apart. In general, the findings indicated that all the Indonesian cattle studied were more closely related, except for the crossbred cattle, which showed a separate cluster from other breeds. The results of this study support the findings of Hartatik *et al*. [31] who reported a close genetic relationship between Indonesian local cattle and Bali cattle, which is a domesticated descendant of the wild Banteng (*Bos javanicus*).

## Conclusion

This study demonstrates polymorphisms in the 5’ UTRs of the *HSP70* gene among the seven cattle populations in Indonesia. The SNPs that occur at a moderate frequency should be further investigated in a larger population to unravel the association between the SNPs and thermotolerance in cattle.

## Data Availability statement

The supplementary data can be available from the corresponding author upon a reasonable request.

## Authors’ Contributions

PWP, AA, and ML: Designed the study and collected the samples. AP: Collected samples and performed laboratory analysis. DNHH: Analyzed the data and wrote the manuscript. All authors have read and approved the final manuscript.
